# Organizing long-term follow-up care for pediatric cancer survivors: a socio-ecological approach

**DOI:** 10.3389/fpubh.2025.1524310

**Published:** 2025-03-03

**Authors:** Ekaterina Aleshchenko, Thorsten Langer, Gabriele Calaminus, Judith Gebauer, Enno Swart, Katja Baust

**Affiliations:** ^1^Faculty of Medicine, Institute of Social Medicine and Health Systems Research, Otto von Guericke University, Magdeburg, Germany; ^2^University Hospital of Schleswig-Holstein, Lübeck, Germany; ^3^Department of Pediatric Hematology and Oncology, University Hospital Bonn, Bonn, Germany

**Keywords:** childhood cancer, cancer survivorship, long-term follow-up studies, ecological systems theory, focus groups

## Abstract

This study examines the organization of long-term follow-up care for pediatric cancer survivors through the lens of Bronfenbrenners’ Ecological Systems Theory (EST). Using focus group discussions with survivors and healthcare professionals across Germany, we aimed to identify challenges and facilitators in care provision. Data were gathered during four focus groups, each consisting of 5–9 participants. A multimethods approach was used, employing both inductive and deductive thematic analysis. Results indicated key challenges such as fragmented care during transitions and insufficient offer of psychological support. The application of EST revealed the importance of coordinating care across multiple system levels: microsystem (direct care), mesosystem (coordination between care settings), exosystem (healthcare policies), and macrosystem (cultural attitudes). The study proposes strategies to improve care, such as implementing case managers and introducing culturally sensible long-term follow-up protocols. These findings highlight the complexity of survivorship care and the need for a more integrated approach to meet the evolving needs of survivors after childhood and adolescent cancer.

## 1 Introduction

With survival rates having significantly improved over the past few decades cancer in childhood and adolescence frequently result in late effects, resulting in frequent ongoing contact with healthcare providers (1, 2). These late effects can be severe, leading to multimorbidity and early mortality, and may not manifest until years or even decades after treatment has ended (3). Many pediatric cancer survivors, even years after treatment, experience higher levels of anxiety and mental health issues compared to the general population (4), resulting in increased hospitalization for mental disorders and higher antidepressant use (5, 6). Pediatric cancer survivors, particularly those with CNS tumors, often face social, cognitive, and academic challenges, including peer difficulties and underperformance in school (7). These early issues can lead to long-term effects such as restricted access to higher education, fewer job prospects, and financial struggles in adulthood (8). The growing population of survivors of cancer in childhood or adolescence, who face an ongoing increased risk of these consequences, require comprehensive support measures to address their health and psychological needs (9, 10).

While during acute treatment, multidisciplinary teams in specialized centers ensure comprehensive medical and psychosocial support, providing high-quality care to nearly all childhood and adolescent cancer patients (11, 12), long-term follow-up remains less homogeneous. However, in recent years, some university hospitals in Germany have set up multidisciplinary long-term follow-up clinics that provide specialized, risk-based monitoring for pediatric cancer survivors (13, 14). These appointments are conducted by either a pediatric oncologist (for adolescent patients), an internist (as a rule, for survivors older than 18), or both (for survivors changing from pediatric to adult healthcare facilities^1^). Additionally, survivors are offered a consultation with psychologists and/or social workers regularly after questionnaire-based screening for psychosocial impairments. Although survivors are encouraged to follow these recommendations, only a small proportion benefit from them for various reasons, with adherence declining after the completion of protocol-based follow-up (15, 16).

While many theories on human development over time exist [such as Life Course Theory (17), Vygotsky’s Sociocultural Theory (18)], we propose that Bronfenbrenner’s Ecological Systems Theory (EST) is particularly valuable for understanding a survivor’s functioning within the larger environmental context (19). EST offers a comprehensive view of how different layers of environmental influences—ranging from immediate family to broader societal factors—interact to shape an individual’s development, making it well-suited for understanding the complex dynamics of survivors’ experiences. Its application in pediatric cancer survivorship has already been demonstrated, showing its effectiveness in capturing the multifaceted challenges and support systems that impact survivors’ long-term well-being (20). Conceptually, EST has been employed to emphasize the importance of influences at the environmental level, creating contextual models that help explain various phenomena. Bronfenbrenner characterized the ecological environment’s structure as “a nested arrangement of structures, each contained within the next,” (21) emphasizing that these interconnected layers must be studied as a unified whole to truly grasp the influences on a developing individual. Bronfenbrenner regarded each system as emerging from a setting, which he described as “a place where people can easily engage in face-to-face interaction” (21). At the most basic level of Bronfenbrenners’ nested hierarchy, microsystems are environments where the individual is directly involved, experiences firsthand, and engages in social interactions with others. Surrounding the microsystems are mesosystems, which encompass the interactions between two of the individual’s settings. Exosystems, in which mesosystems are nested, consist of environments that impact the individual indirectly, as the individual does not actively participate in them. Lastly, macrosystems, which encompass exosystems, represent the broader cultural influences or ideologies that have far-reaching effects on the individual. Beyond the four core systems of ecological systems theory, Bronfenbrenner later introduced the chronosystem, which represents changes or continuity over time and affects all the other systems (22).

While a few studies have examined the organization of long-term follow-up care for childhood and adolescent cancer in Germany [e.g., (14, 23)], a comprehensive approach that integrates the perspectives of survivors, their families, and healthcare providers is still lacking. To address this gap, this study aimed to discuss survivorship pathways, described based on the experiences of survivors and their informal caregivers, with healthcare providers. The goal is to propose comprehensive improvements to the current organization of long-term follow-up care. This analysis is part of the broader VersKiK project, which explores the (long-term) effects of childhood and adolescent cancer, adherence to (long-term) follow-up guidelines, and the actual needs of survivors and their informal caregivers. A detailed description of the VersKiK project’s overall design and the methodology of this study can be found in separate publications (24, 25).

## 2 Materials and methods

The VersKiK project was approved by the Ethics Committee Otto von Guericke University on 2.07.2021 (103/21), by the Ethics Committee of Johannes Gutenberg University Mainz on 16.06.2021 (2021-16035), by the Ethics Committee University of Lübeck on 10.11.2021 (21-451), by the Ethics Committee University of Hospital Bonn on 28.02.2022 (05/22). For each part of the qualitative study—including the case study development based on participant observations and interviews with survivors and their parents, as well as the focus groups—separate written informed consent was approved by the Ethics Committees (25).

Healthcare professionals’ and survivors representatives’ experiences in participation in long-term follow-up care were explored through focus groups (26). Focus group methodology was selected because it can offer in-depth insights into areas with limited existing data or knowledge (27). We conducted four focus groups across various regions of Germany between December 2023 and February 2024. Each group comprised between 5 and 9 participants. The detailed characteristics of the focus group participants are outlined in Table 1. We recruited participants purposively (28) through university hospitals attending the project. The participants represented diverse disciplines involved in follow-up care, including pediatric oncologists, psychologists, gynecologists, nurses, further professionals specializing in long-term survivor care, such as sports therapists and nutritionists, and patient advocates. The participants in each focus group were from different organizations and did not have any dependent relationships (e.g., patient- health care provider) to prevent potential power dynamics. They also received no compensation for their participation.

**Table 1:** Focus groups participants.

Focus group	Number of participants	Participants
1	5	Pediatric oncologist, director of rehabilitation clinic, psychologist, sports therapist, study nurse, internist
2	6	Survivor, two pediatric oncologists, internist, social worker, pediatric endocrinologist
3	7	Pediatric oncologist, two social workers, sports therapist, survivor, gynecologist, nurse,
4	9	Two survivors, sports therapist, gynecologist, nurse, social worker, pediatric oncologist, internist, psychologist

Two case studies, illustrating the survivorship pathways of an adult and a transitional childhood cancer survivors, were used to stimulate focus group discussions. The development of these case studies is explained in another source (29). Before the focus groups, all participants received the case studies for review. EA, a psychologist and skilled moderator, facilitated the sessions, with a scribe present to take notes. Focus groups lasted between 90 and 120 min and were audio-recorded. Informed consent was obtained from all participants.

We analyzed the focus group notes using an adaptive theory approach (30), which integrates both inductive and, to a smaller extent, deductive methods. This approach allowed us to examine the data to confirm, challenge, and investigate previously identified theoretical concepts and connections, while also uncovering new concepts and relationships. An inductive approach was applied for the initial analysis of focus groups notes. Notes were read multiple times to develop a thorough understanding of the data, with early impressions recorded. Open coding was employed to create and refine initial themes. EA performed manual line-by-line coding of the notes to enhance reflexivity, and then results were reviewed and compared for consistency and relevance by KB, a researcher and psychotherapist directly involved in practical (long-term) follow-up care. The themes were deductively compared with those used for case study development. The analyses results are presented in Table 2.

**Table 2:** Application of Bronfenbrenner’s Ecological Systems Theory: focus groups discussions.

Model dimensions	Themes, addressed in a case study	Focus groups impressions
Case Study 1. Adult Woman (35 years old, diagnosed with lymph node cancer)		
Microsystem	Interactions with healthcare providers Family and peer interactions	Challenges of navigating long-term follow-up care when healthcare providers change frequently, causing a loss of trust and a sense of instability; The importance of family support, noting that survivors often rely on family members to manage healthcare needs
Mesosystem	Interactions between family and healthcare providers Family and work interactions	Poor communication between healthcare providers and families often leads to fragmented care, with survivors having to manage multiple roles without sufficient support; Difficulties that survivors face when trying to balance work, family, and long-term follow-up care, with inadequate support from employers and the healthcare system exacerbating these challenges
Exosystem	Healthcare policies and work-related issues Impact of work on care	Survivors needs, guideline based diagnostic and treatment is not fully covered by statutory health insurance, leading to stress and disrupted care routines; Rigid work schedules and the lack of employer understanding can interfere with necessary medical appointments
Macrosystem	Broader cultural and societal factors, incl. fertility issues and societal expectations Social stigma, e.g., societal perception of being “healthy” post-treatment	Survivors often feel pressured to conform to societal expectations of health, leading to reluctance in seeking necessary support; The perception that survivors should be “healthy” after treatment, and thus not in need of continued care, was a significant issue discussed
Chronosystem	Impact of time and life transitions, leading to neglected her long-term follow-up care Changes in Healthcare Needs with increasing age and survival period	As survivors age and encounter new life stages, their healthcare needs evolve, often without adequate support from the system; Survivors’ needs change over time, and the healthcare system often fails to adapt, particularly in providing long-term psychological and emotional support
Case Study 2. Young Man (18 years old, diagnosed with a brain tumor)		
Microsystem	Interactions with healthcare providers, incl. the absence of a consistent healthcare provider at the immediate neighborhood Family and peer interactions, incl. dependency issues	Comprehensive long-term follow-up care is often lacking, leaving survivors without the necessary support to manage ongoing health issues; Over-reliance on family members can hinder the development of autonomy in young survivors
Mesosystem	Interactions between family and healthcare providers Education and healthcare system interactions	An issue of poor integration between different care systems; Insufficient collaboration between educational institutions and healthcare providers, leading to clashes in survivor’s schedules
Exosystem	Healthcare policies and school-related issues, incl. the lack of effective coordination between different systems Impact of healthcare system, incl. delays in care provision	External factors, such as inefficient healthcare systems and unsupportive school environments, significantly impact survivors’ well-being; Systemic barriers to accessing necessary healthcare services, particularly in specialized areas
Macrosystem	Broader cultural and societal factors, incl. stigma and societal pressures Social norms and expectations, e.g., struggle with fitting into social settings	Survivors often feel marginalized due to their disabilities, which can lead to social isolation and decreased self-esteem; Societal expectations can negatively affect survivors’ ability to integrate into social and educational settings
Chronosystem	Impact of time and life transitions, e.g., request for independence in managing health condition Long-term effects of treatment, challenging more with transition to adulthood medical facilities	The transition from adolescence to adulthood is particularly challenging for survivors, especially when they face additional health-related responsibilities; The healthcare system often fails to provide adequate long-term support, leaving survivors to manage these issues largely on their own

## 3 Results

### 3.1 Application of Bronfenbrenners’ Ecological Systems Theory on long-term follow-up care provision: focus groups’ notes analyses

**Figure 1:**
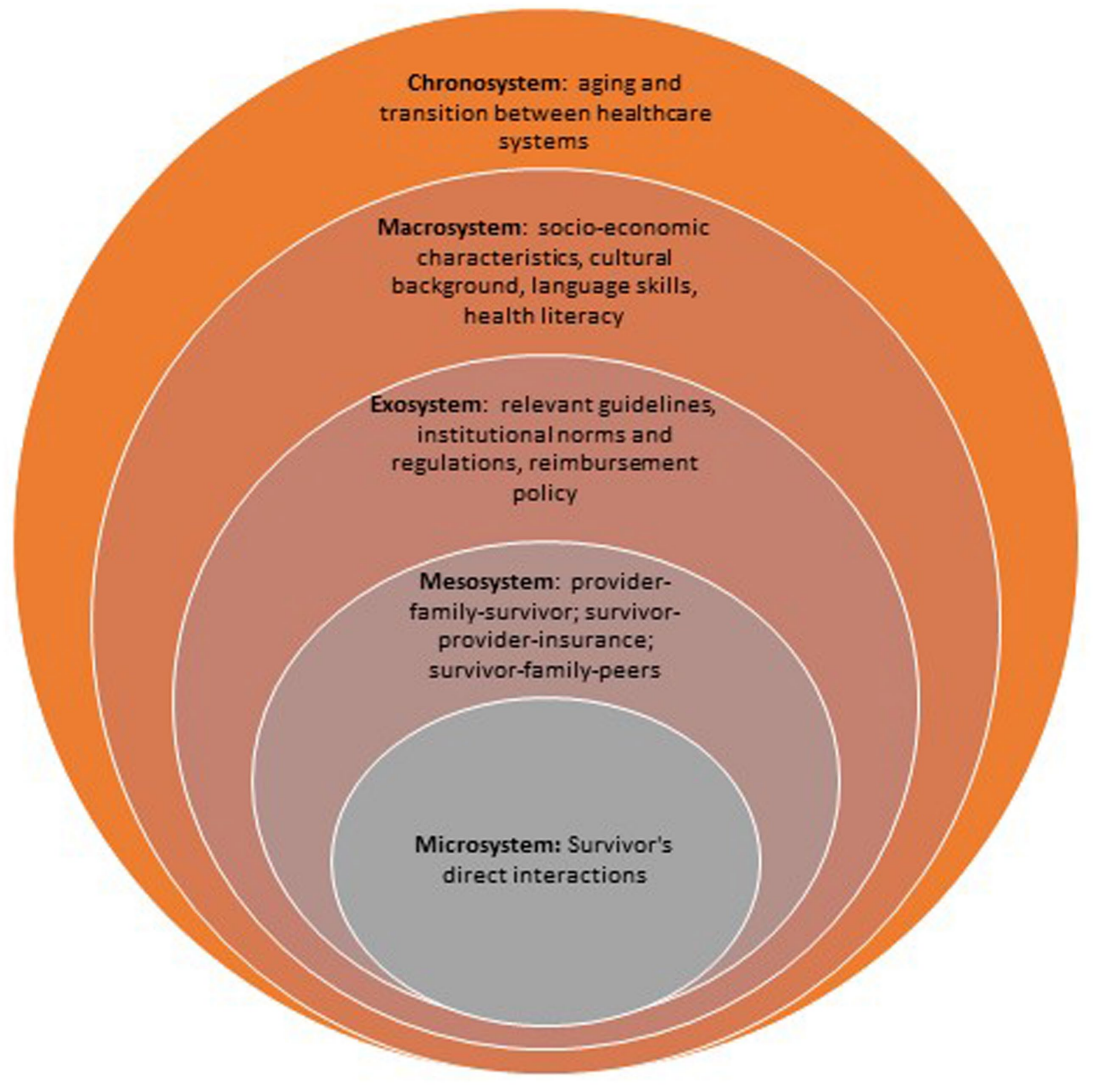
Application of Bronfenbrenner’s Ecological System Theory in pediatric cancer follow-up care.

We propose the following application of EST to the organization of pediatric cancer long-term follow-up care, as illustrated in Figure 1, for further analysis. Each layer is interrelated, highlighting the intricate nature of coordinating long-term follow-up care for pediatric cancer survivors:

**Microsystem**: Represents the direct environment of the survivor, including family, healthcare providers, and immediate care settings. The focus here would be on fostering supportive relationships between the survivor, their family, and healthcare professionals.

**Mesosystem**: This connects different microsystems, like communication and collaboration between family members and healthcare providers, or between different medical institutions. Coordination among these groups is vital for continuous and multidisciplinary long-term follow-up care.

**Exosystem**: Involves broader influences, such as healthcare policies, insurance systems, and parental workplaces that indirectly impact the survivors’ care. Effective policies and workplace flexibility for both adult survivors and informal caregivers could improve access to long-term follow-up services.

**Macrosystem**: Encompasses cultural beliefs, societal norms, and laws that affect healthcare delivery, especially in the context of pediatric cancer survivorship. It involves the broader societal attitudes and regulations that shape how care is provided.

**Chronosystem**: Addresses changes over time, such as the survivors’ development and evolving health needs, ensuring that long-term follow-up care adapts as the survivor transitions from pediatric to adult care systems.

Further, we discuss the defined themes according to the EST dimensions. The comprehensive analyses including themes coded in case studies and themes specified during focus group discussions are presented in Table 2.

#### 3.1.1 Microsystem

##### 3.1.1.1 Healthcare providers and survivors

Direct interactions between survivors and healthcare providers are crucial. Challenges such as the lack of continuity in care, particularly during the transition from pediatric to adult care, create significant barriers for survivors. Survivors often struggle with fragmented care, requiring multiple referrals from general practitioners, which they must organize themselves. If psychological support is (as usually) not available, this further exacerbates the emotional burden of transition. For example, one of the participating survivors mentioned that she only learned very late about the importance of long-term follow-up care. Other doctor underlines the relevance of information on late effects during acute treatment addressing both patients and parents, especially related to reproduction medicine. Similarly, other survivors noted feeling unsupported due to inconsistent medical care. One of them emphasized the benefits of an initial consultation leading to a personalized follow-up plan, including a mandatory psychological consultation, which might help feel understood and less isolated. A pediatric oncology nurse noted that children often depend on their parents for follow-up decisions, leading to discrepancies in engagement levels among families. Additionally, as mentioned by another survivor, access to follow-up care remains limited, with survivors facing long travel distances to reach specialized clinics.

##### 3.1.1.2 Family

Family support plays a pivotal role in a survivor’s microsystem. Parental involvement in decision-making and long-term follow-up care is crucial during adolescence. However, as survivors transition into adulthood, they must take on greater responsibility for their healthcare. This transition can be emotionally challenging, especially when it requires survivors to recount their entire medical history and reestablish medical care in new healthcare facilities. One of the survivors recalled that during his illness, his school received information from a hospital pedagogue to help his classmates understand his situation and thereby facilitating his independent communication about health issues. Similarly, another one noted that during his treatment, most information was conveyed to his parents, leaving him with limited agency over his care. Overprotective parents can further complicate the transition to independence, making it difficult for survivors to take charge of their health. The discussion highlighted that while family involvement is essential, empowering adolescents early on to manage their healthcare journey is equally important.

#### 3.1.2 Mesosystem

##### 3.1.2.1 Family and healthcare providers

The interaction between families and healthcare providers is often marked by communication challenges, particularly in coordinating care during transitions. Focus groups’ participants noted that parents sometimes feel uninformed or overburdened, affecting their ability to effectively support the survivor. For instance, a doctor in a long-term follow-up clinic stressed that families should receive continuous psychological support both during and after the acute phase. However, due to inconsistent care structures, some survivors face inner barriers to participating in long-term follow-up. A social worker highlighted that many families struggle to justify continued disability benefits or special accommodations since survivors no longer appear acutely ill, despite ongoing health challenges. Similarly, a psychotherapist pointed out that professionals often assume that families will independently take on the responsibility of long-term care management, leading to significant disparities in follow-up adherence.

##### 3.1.2.2 Healthcare system interactions

Significant gaps exist in communication between different healthcare providers, especially during the transition from pediatric to adult healthcare system. This lack of integration leads to fragmented care, making it difficult for survivors to navigate long-term follow-up care. A doctor in a long-term follow-up clinic pointed out that patients with a high burden of disease are more likely to seek follow-up care, while those without acute symptoms tend to avoid it and thereby reducing the chance to detect new late effects in due time. Additionally, survivors mentioned that they experience difficulty navigating the system, as they must advocate for themselves and obtain multiple referrals. A pediatric oncologist stressed the importance of standardized and comprehensive information about long-term follow-up after standard post-treatment care to facilitate smoother transitions. Furthermore, she emphasized the difficulty in integrating routine follow-ups into university clinics due to reimbursement peculiarities. Other medical experts stressed the importance of long-term multidisciplinary care, provided in university settings and supported by a dedicated payment system.

#### 3.1.3 Exosystem

##### 3.1.3.1 Healthcare policies

Healthcare policies present significant challenges, such as insufficient funding for long-term follow-up care and a lack of implementation of standardized long-term follow-up care guidelines. For instance, a gynecologist emphasized the importance of fertility preservation counseling before cancer treatment. However, she noted that inadequate awareness among healthcare providers means that many survivors miss the opportunity to discuss fertility options before undergoing treatment. Additionally, funding issues make it difficult to provide continuous psychosocial support. A psychologist suggested that automatic appointment scheduling for follow-up care could help ensure that survivors are aware of available services rather than relying on self-initiation. A pediatric oncologist proposed a modular system where a basic check-up would be standardized, with additional services tailored to specific risks.

##### 3.1.3.2 Work-related issues

Survivors and their families often face difficulties in balancing work and school/study commitments with long-term follow-up care appointments. The rigid structure of healthcare appointments exacerbates this issue. One of the survivors mentioned that many survivors have to travel long distances to reach specialized follow-up clinics, making it hard to fit these appointments into their work or study schedules. Additionally, a social worker noted that some survivors hesitate to disclose their health history in professional settings due to stigma, making it challenging to request workplace accommodations. Another survivor highlighted how some employers show little understanding for necessary follow-up appointments, particularly for individuals with physical limitations, making comprehensive aftercare difficult to maintain.

#### 3.1.4 Macrosystem

##### 3.1.4.1 Cultural perceptions

Broader societal and cultural factors, such as the stigma associated with cancer survivorship and psychological care, significantly influence survivors’ willingness to engage in long-term follow-up care. The reluctance to address mental health issues is particularly noted in certain cultural contexts, where discussing cancer is often taboo. A social worker observed that many survivors avoid social workers because they do not want to acknowledge their ongoing health issues, fearing judgment or pity. One of the survivors also mentioned that survivors often have to justify their need for follow-up care because others perceive them as fully recovered. Additionally, a pediatric oncologist explained that male adolescent patients often reject discussions about fertility preservation due to feelings of embarrassment, which leads to missed opportunities for sperm banking. A social worker further noted that working with translators can sometimes result in critical fertility-related information being lost or deliberately withheld in more conservative cultural backgrounds.

##### 3.1.4.2 Societal support systems

The general societal structure, including the availability of support groups and social networks, is crucial for survivors but often inadequate. One of the survivors, who has been part of a survivor group for more than 20 years now, highlighted how peer connections helped him navigate the long-term effects of cancer. However, he also noted that many survivors remain unaware of these support structures. A sports scientist emphasized the benefits of physical activity for survivors but pointed out that there are few tailored exercise programs, particularly in rural areas. However, a pediatric oncologist noted that joining such groups requires significant courage. Online options, such as video tutorials or anonymous forums, were proposed as an alternative, though concerns regarding misinformation were raised. Healthcare professionals advocated for objective, medically vetted (online-) resources instead of relying on social media.

#### 3.1.5 Chronosystem

##### 3.1.5.1 Life transitions

Time and life transitions significantly impact survivors’ experiences, especially during the shift from pediatric to adult care. Focus groups emphasized the need for more structured and supportive transitions to mitigate the psychological and logistical challenges that arise during this period. Survivors often struggle with navigating complex medical systems on their own, which can complicate their transition. For example, a pediatric oncologist suggested that survivors should receive standardized information about long-term follow-up care at the end of their standard post-treatment phase. A psychologist highlighted the emotional difficulty survivors face when having to rebuild their medical care network after moving or becoming independent. A gynecologist added that interdisciplinary transition meetings could ease this burden, and a pediatric oncologist advocated for structured information pathways throughout the entire treatment journey, ensuring that survivors receive consistent guidance.

##### 3.1.5.2 Changes in healthcare needs with increasing age and survival period

As survivors age, their healthcare needs change, and the healthcare system often fails to adapt to these evolving needs, particularly in providing long-term support, including psychological, social, and legal counseling. Focus groups noted the need for a more responsive system. A psychologist emphasized that medical professionals must remain vigilant to avoid attributing all health issues to a past cancer diagnosis, potentially overlooking other conditions. One of the survivors stressed the importance of health literacy, as she only discovered long-term follow-up care through her individual research. Another survivor proposed the development of a digital app, that would streamline follow-up care by tracking past consultations and providing reminders for necessary check-ups. This concept aligns with the broader discussion on digital health solutions as a means of improving continuity of care, and the focus on simplifying administrative procedures and better educating survivors on the importance of follow-up care (31).

### 3.2 Barriers and facilitators in long-term follow-up care provision

We also examined the barriers and facilitators identified in focus groups separately. Table 3 highlights both the barriers and facilitators in the organization of long-term follow-up care for pediatric cancer survivors.

**Table 3:** Long-term follow-up care provision: barriers and facilitators.

Barriers	Facilitators
Lack of continuity in care leads to fragmented care and loss of trust (esp. during transition)	Family involvement as a key facilitator, particularly during the transition from pediatric to adult care
Inadequate coordination between systems: survivors often have to navigate long-term follow-up care on their own, leading to gaps in care	The importance of having structured and coordinated multidisciplinary care programs that integrate different aspects of a survivor’s care
Psychological and emotional barriers, e.g., anxiety and depression, may not be not adequately addressed in long-term follow-up care	Access to information and resources: clear, accessible information about their long-term follow-up care options and the importance of continued care
Healthcare system limitations, e.g., reimbursement issues, lack of standardized care protocols, and insufficient offer of psychological services	Timely information: detailed information on all relevant aspects of follow-up provided on the early stages/before acute cancer treatment
Socioeconomic and cultural barriers: Survivors from lower socioeconomic backgrounds or certain cultural groups may face additional barriers, such as language barriers or lack of access to specialized services	Psychological support services, including social law support, significantly improve survivors’ quality of life and their engagement with long-term follow-up care

Key **barriers** include a lack of continuity in care, particularly during the transition from pediatric to adult care, leading to fragmented care and a loss of trust. Survivors often face inadequate coordination between different systems, forcing them to navigate long-term follow-up care on their own, which results in gaps in care. Additionally, psychological and emotional issues, such as anxiety and depression, are often not sufficiently addressed. The healthcare system itself poses challenges, including issues with reimbursement, a lack of standardized care protocols, and insufficient offer of psychological services. Socioeconomic and cultural factors also present significant hurdles, with survivors from lower socioeconomic backgrounds or culturally and linguistically diverse groups facing additional challenges, such as language barriers and limited access to specialized services.

On the other hand, **facilitators** for improving long-term follow-up care include strong family involvement, which is particularly crucial during the transition from pediatric to adult care. Structured and well-coordinated multidisciplinary care programs that integrate various aspects of the survivors’ care also play an important role in ensuring continuity and quality of care. Access to clear and accessible information about long-term follow-up care options is another facilitator, as it helps survivors understand their options and the importance of continued care. Furthermore, an increased need for information was recognized, indicating that families should be provided with detailed knowledge about all relevant aspects of follow-up care starting during acute therapy through interdisciplinary cooperation. Specifically, collaboration with reproductive medicine was noted as a key example of this interdisciplinary approach. Lastly, the availability of psychological support services, including social and legal counseling, significantly enhances survivors’ quality of life and increases their engagement with long-term follow-up care.

### 3.3 Suggested improvement measures for long-term follow-up care organization

To enhance the quality of long-term follow-up care for pediatric cancer survivors, we suggest several key improvement measures based on our analyses. These measures focus on addressing the identified barriers and enhancing the facilitators of care provision across different systems.

#### 3.3.1 Providing continuity of care

Continuity of care is crucial in ensuring that survivors receive consistent support throughout their healthcare journey. One effective strategy is the implementation of dedicated case managers. These professionals would be assigned to each survivor to coordinate care across various providers and systems, ensuring seamless transitions, particularly from pediatric to adult care. Additionally, establishing long-term follow-up programs that are easily accessible, regardless of geographic location or provider changes, can help maintain consistent care for survivors throughout their lives.

#### 3.3.2 Improving coordination between systems

Coordinating healthcare providers, families, and other support systems is essential for effective long-term follow-up care. Developing multidisciplinary care networks that link various stakeholders, including healthcare professionals, schools, employers, and families, would ensure that all aspects of a survivor’s life are considered in their care plan. Furthermore, regular cross-disciplinary communication between care providers—such as oncologists, psychologists, and general practitioners—should be facilitated to provide a more holistic approach to survivor care. This would reduce fragmented care and improve outcomes.

#### 3.3.3 Addressing psychological and emotional needs

To ensure survivors’ mental well-being, it is essential to integrate routine psychological assessments into long-term follow-up care. Early identification and intervention for mental health challenges would significantly improve survivors’ overall quality of life. Offering on-site or telehealth psychological services would make support more accessible. Additionally, fostering peer support networks and survivor support groups would help to address feelings of isolation and reduce stigma, providing a sense of community and shared experience.

#### 3.3.4 Strengthening the healthcare system

To address inconsistencies in care, general healthcare professionals’ awareness of long-term follow-up care should be improved. It would contribute to overall better and harmonized long-term follow-up care, regardless of location, including long-term monitoring, psychological support, and effective coordination between specialists. Increasing funding and resources for long-term care services, particularly in underserved areas, is also essential to reduce disparities in care quality and accessibility.

#### 3.3.5 Addressing socioeconomic and cultural barriers

Culturally sensitive care models should be developed to accommodate the diverse backgrounds of survivors. This could involve providing translation services, culturally tailored health information, and outreach programs to engage survivors from various cultural and socioeconomic groups. Educating employers on the importance of long-term follow-up care for cancer survivors could also help create more supportive work environments for these individuals.

The suggested measures are specifically tailored to improve long-term follow-up care for pediatric cancer survivors in Germany, addressing the unique challenges and systems in place within the country. However, some of these measures, such as the development of integrated care networks, the implementation of dedicated case managers, and the enhancement of culturally sensitive care, could be transferable to broader international contexts, such as other European countries or regions with similar healthcare structures.

## 4 Discussion

The aim of this study was to explore the challenges and facilitators in the organization of (long-term) follow-up care for pediatric cancer survivors by applying Bronfenbrenner’s Ecological Systems Theory. Through focus group discussions with survivors and healthcare professionals, this research sought to identify critical areas for improvement and offer practical recommendations to enhance the continuity, coordination, and quality of care across various levels of the healthcare system. The findings of this study underscore the complexity of providing effective (long-term) follow-up care for childhood and adolescence cancer survivors, particularly when considering the broad range of physical, psychological, and social challenges they face. By applying Bronfenbrenners’ Ecological Systems Theory (EST), we examined the multiple environmental layers influencing the care process, highlighting critical gaps in continuity, coordination, and psychosocial support.

One of the most pressing issues identified in the present study is the fragmentation of care, particularly during the transition from pediatric to adult healthcare systems. This transition often leads to a breakdown in communication between providers and a loss of trust, leaving survivors without the consistent support they require. These results are consistent with prior research showing that survivors’ strong connections to pediatric healthcare facilities can influence the transition process and undermine their confidence in the quality of care delivered by adult healthcare providers (32, 33). Focus group participants proposed the introduction of dedicated case managers as part of a multidisciplinary long-term follow-up team, which could be crucial in closing this gap by providing survivors with coordinated care across various settings and offering a reliable and stable contact person they can trust. Earlier studies have demonstrated that appointing a nurse as a case manager can enhance multidisciplinary collaboration and promote the delivery of integrated care (34, 35). Our findings align with the recommendations proposed by the PanCareSurFup Guidelines Working Group (36), particularly regarding the necessity of structured care, coordinated by cancer survivorship expert centers, and highlighting the role of multidisciplinary teams in delivering follow-up care.

Moreover, the need for improved communication between healthcare providers and families emerged as another significant challenge. Survivors and their caregivers often feel uninformed or unsupported due to inconsistent information flow between medical teams. In line with the findings of the scoping review by Wong et al. (37), focus group participants suggested the development of integrated care networks that promote regular, multidisciplinary communication. Such networks could help address these challenges and lead to more comprehensive, patient-centered care. They also emphasized the need to raise awareness among general healthcare providers regarding the long-term follow-up care of pediatric cancer survivors. Similarly, past research has underscored the vital role of primary care providers in offering appropriate screening and effective treatment options, addressing the needs of both cancer survivors and their families (38). Another potential solution involves implementing digital tools to assist survivors and their families during the periods between follow-up appointments, which tend to become longer as more time passes since the completion of acute treatment. Similarly, a feasibility study by Demoor-Goldschmidt et al. demonstrated the strong potential of a digital intervention to support survivors and their families in long-term follow-up care (39).

Psychosocial support is also a key area where improvements are necessary. The mental health challenges faced by survivors, such as anxiety and depression, are frequently under-addressed in long-term follow-up care (40). In line with Barrett et al. (2), focus group participants suggested that incorporating regular psychological assessments and offering access to mental health services, either in person or via telehealth, would help meet these unaddressed needs. Beyond mental health, many survivors face significant social challenges, particularly in navigating life after cancer. These can include issues related to employment, education, and social integration, especially for those dealing with disabilities resulting from their treatment (7, 8). Focus group findings highlighted the necessity of both structural and functional social support, as outlined by Deegan et al. (41). On one hand, offering legal advice alongside psychosocial support is vital to assist survivors in understanding their rights and obtaining disability benefits or accommodations in work and educational settings. On the other hand, peer support networks and survivor groups can help alleviate feelings of isolation and build a sense of community, allowing survivors to share their experiences and support each other through the long-term challenges of life after cancer. These findings are consistent with a study by Matsui et al., which found that “having a confidant” and “forming friendships with other AYA (adolescent and young adult) patients” were positively linked to posttraumatic growth (42).

Culturally sensitive care models are another area of focus. Our study revealed that survivors from diverse backgrounds often face additional barriers, such as language challenges and cultural stigmas surrounding cancer and/or mental health care. Similarly, an integrative review by Yeom et al. identified cultural factors such as values, beliefs, fatalism, social norms, faith or religion, gender roles, and traditions as influencing self-care, which is a key component of long-term follow-up care for cancer survivors (43). Tailoring long-term follow-up care to be more culturally responsive, through translation services and outreach programs, would improve access and engagement for these populations.

This study has several strengths. The use of Bronfenbrenner’s Ecological Systems Theory (EST) provides a comprehensive framework for understanding the multi-level influences on pediatric cancer survivors’ long-term follow-up care. The inclusion of multiple stakeholders—survivors, caregivers, and healthcare providers—enriches the data and offers a broader perspective on care challenges and facilitators. Additionally, the study proposes practical solutions, such as early information on late effects, case managers, and integrated care networks, which can enhance follow-up care. A key contribution is its focus on the critical transition from pediatric to adult care, addressing a common gap in continuity.

At the same time, the study has some limitations. As it is based on the German healthcare system, findings may need adaptation to special settings of other healthcare systems. While the qualitative approach allows for in-depth exploration, focus group discussions may reflect group dynamics, though efforts were made to balance participant contributions. Retrospective reflections provided valuable insights into survivorship experiences rather than factual recall.

Future research should explore the transferability of these findings to different healthcare systems and complement them with quantitative studies for a broader perspective. Further evaluation of proposed interventions, such as case managers and multidisciplinary care models, is needed to assess their impact. Longitudinal studies could also provide deeper insights into the evolving needs of survivors, ensuring follow-up care remains responsive over time.

## 5 Conclusion

This study emphasizes the importance of establishing structured, coordinated, multidisciplinary care systems that cater to the complex needs of pediatric cancer survivors. Key interventions should aim to strengthen continuity of care, enhance cross-system communication, incorporate culturally sensitive components into care, and offer psychosocial support. Implementing these measures could significantly improve survivors’ long-term health outcomes and quality of life.

## Data Availability

The raw data supporting the conclusions of this article will be made available by the authors without undue reservation.

## Footnotes

^1^Available in a limited number of university hospitals.

## References

[1] Miller KD Pandey M Jain R Mehta R. Cancer survivorship and models of survivorship care: a review. Am J Clin Oncol. (2015) 38:627–33. doi: 10.1097/COC.0000000000000153, PMID: 25635609

[2] Barrett PM Mullen L McCarthy T. Enduring psychological impact of childhood cancer on survivors and their families in Ireland: a national qualitative study. Eur J Cancer Care (Engl). (2020) 29:e13257. doi: 10.1111/ecc.13257, PMID: 32537764 PMC7988562

[3] Oeffinger KC Robison LL. Childhood cancer survivors, late effects, and a new model for understanding survivorship. JAMA. (2007) 297:2762–4. doi: 10.1001/jama.297.24.2762, PMID: 17595279

[4] Fidler MM Frobisher C Hawkins MM Nathan PC. Challenges and opportunities in the care of survivors of adolescent and young adult cancers. Pediatr Blood Cancer. (2019) 66:e27668. doi: 10.1002/pbc.27668, PMID: 30815985

[5] Lund LW Winther JF Cederkvist L Andersen KK Dalton SO Appel CW. Increased risk of antidepressant use in childhood cancer survivors: a Danish population-based cohort study. Eur J Cancer (Oxford, England: 1990). (2015) 51:675–84. doi: 10.1016/j.ejca.2015.01.001 25677304

[6] Johannsdottir IM Karlstad Ø Loge JH Fosså SD Kiserud C Skurtveit S. Prescriptions of antidepressants to survivors of cancer in childhood, adolescence, and young adulthood: a population-based study. J Adolesc Young Adult Oncol. (2017) 6:120–6. doi: 10.1089/jayao.2016.0041, PMID: 27841952

[7] Park M Park HJ Lee JM Ju HY Park BK Yu E-S. School performance of childhood cancer survivors in Korea: a multi-institutional study on behalf of the Korean Society of Pediatric Hematology and Oncology. Psycho-Oncology. (2018) 27:2257–64. doi: 10.1002/pon.4819, PMID: 29927510

[8] Nathan PC Henderson TO Kirchhoff AC Park ER Yabroff KR. Financial hardship and the economic effect of childhood cancer survivorship. J Clin Oncol. (2018) 36:2198–205. doi: 10.1200/JCO.2017.76.4431, PMID: 29874136

[9] Skinner R Wallace WHB Levitt G. Long-term follow-up of children treated for cancer: why is it necessary, by whom, where and how? Arch Dis Child. (2007) 92:257–60. doi: 10.1136/adc.2006.095513, PMID: 17337686 PMC2083428

[10] Gebauer J Skinner R Haupt R Kremer L van der Pal H Michel G. The chance of transition: strategies for multidisciplinary collaboration. Endocr Connect. (2022) 11. doi: 10.1530/EC-22-0083, PMID: 35900792 PMC9422248

[11] Rossig C Juergens H Schrappe M Moericke A Henze G von Stackelberg A. Effective childhood cancer treatment: the impact of large scale clinical trials in Germany and Austria. Pediatr Blood Cancer. (2013) 60:1574–81. doi: 10.1002/pbc.24598, PMID: 23737479

[12] Schröder HM Lilienthal S Schreiber-Gollwitzer BM Griessmeier B Leiss U. L - Psychosoziale versorgung in der pädiatrischen onkologie und hämatologie (S3) In: Wirth S Creutzig U Krauspe R Lehrnbecher T Mentzel H-J Niehues T, editors. Leitlinien Kinder- und Jugendmedizin. Munich: Urban & Fischer (2015). L.17–34.

[13] Gebauer J Lehnert H Schmid SM Spix C Stein A Langer T. Spätfolgen einer Krebsbehandlung im Kindes- und Jugendalter: Eine Herausforderung für die Transitionsmedizin. Internist (Berl). (2018) 59:1157–62. doi: 10.1007/s00108-018-0496-0, PMID: 30229367

[14] Gebauer J Baust K Bardi E Grabow D Stein A van der Pal HJ. Guidelines for long-term follow-up after childhood cancer: practical implications for the daily work. Oncol Res Treat. (2020) 43:61–9. doi: 10.1159/000504200, PMID: 31931503

[15] Ekaterina A Thorsten L Gabriele C Juliane G Kathrin H Pietro T. Follow-up care needs and motivational factors for childhood cancer survivors and their parents in Germany. Sci Rep. (2025) 15:972. doi: 10.1038/s41598-024-84156-y, PMID: 39762346 PMC11704210

[16] Michel G Kuehni CE Rebholz CE Zimmermann K Eiser C Rueegg CS. Can health beliefs help in explaining attendance to follow-up care? The Swiss childhood cancer survivor study. Psychooncology. (2011) 20:1034–43. doi: 10.1002/pon.1823, PMID: 20687196

[17] Hutchison ED. Life course theory In: Levesque RJR, editor. Encyclopedia of adolescence. New York, NY: Springer (2011). 1586–94.

[18] Vygotsky LS. Mind in society: development of higher psychological processes. Boston: Harvard University Press (1978).

[19] Bronfenbrenner U. Ecology of human development: experiments by nature and design. Cambridge: Harvard University Press (2009).

[20] Schwartz LA Tuchman LK Hobbie WL Ginsberg JP. A social-ecological model of readiness for transition to adult-oriented care for adolescents and young adults with chronic health conditions. Child Care Health Dev. (2011) 37:883–95. doi: 10.1111/j.1365-2214.2011.01282.x, PMID: 22007989

[21] Bronfenbrenner U. The ecology of human development: experiments by nature and design. Boston: Harvard University Press (1979).

[22] Bronfenbrenner U. Ecology of the family as a context for human development: research perspectives. Dev Psychol. (1986) 22:723–42. doi: 10.1037/0012-1649.22.6.723

[23] Gebauer J Hilgendorf I Koch B Langer T. Neue Strukturen in der Betreuung und Nachsorge junger Krebspatienten. Oncol Res Treat. (2019) 42:21–6. doi: 10.1159/000500818

[24] Aleshchenko E Swart E Spix C Voigt M Trocchi P Langer T. Long-term care, care needs and wellbeing of individuals after cancer in childhood or adolescence (VersKiK): study protocol of a large scale multi-methods non-interventional study. BMC Health Serv Res. (2022) 22:1176. doi: 10.1186/s12913-022-08549-3, PMID: 36127717 PMC9487026

[25] Aleshchenko E Swart E Voigt M Langer T Calaminus G Glogner J. VersKiK qualitative study design: actual follow-up needs of paediatric cancer survivors, their informal caregivers and follow-up stakeholder perceptions in Germany. BMJ Open. (2024) 14:e072860. doi: 10.1136/bmjopen-2023-072860, PMID: 38326270 PMC10860087

[26] Bryman A. Social research methods. 5th ed. Oxford: Oxford University Press (2015).

[27] Adler K Salanterä S Zumstein-Shaha M. Focus group interviews in child, youth, and parent research: an integrative literature review. Int J Qual Methods. (2019) 18:1609406919887274. doi: 10.1177/1609406919887274

[28] Polit DF Beck CT. Nursing research: Generating and assessing evidence for nursing practice. Tenth ed. Philadelphia: Wolters Kluwer (2017).

[29] Aleshchenko E Langer T Calaminus G Glogner J Gebauer J Swart E. Case study in VersKiK: a methodological approach for studying pediatric cancer survivors’ pathways. BMC Med Res Methodol. 10.1186/s12874-025-02723-x PMC12696934 41331574

[30] Bulmer M Layder D. Sociological practice: linking theory and social research. Can J Sociol. (2001) 26:125. doi: 10.2307/3341515

[31] Filbert A-L Kremer L Ladenstein R Chronaki C Degelsegger-Márquez A van der Pal H. Scaling up and implementing the digital survivorship passport tool in routine clinical care - the European multidisciplinary PanCareSurPass project. Eur J Cancer. (2024) 202:114029. doi: 10.1016/j.ejca.2024.114029, PMID: 38513384

[32] Otth M Denzler S Koenig C Koehler H Scheinemann K. Transition from pediatric to adult follow-up care in childhood cancer survivors-a systematic review. J Cancer Surviv. (2021) 15:151–62. doi: 10.1007/s11764-020-00920-9, PMID: 32676793

[33] Marchak JG Sadak KT Effinger KE Haardörfer R Escoffery C Kinahan KE. Transition practices for survivors of childhood cancer: a report from the children's oncology group. J Cancer Surviv. (2023) 17:342–50. doi: 10.1007/s11764-023-01351-y, PMID: 36870037 PMC9984742

[34] Karam M Chouinard M-C Poitras M-E Couturier Y Vedel I Grgurevic N. Nursing care coordination for patients with complex needs in primary healthcare: a scoping review. Int J Integr Care. (2021) 21:16. doi: 10.5334/ijic.5518, PMID: 33776605 PMC7977020

[35] Signorelli C Wakefield CE Johnston KA Fardell JE McLoone JK Brierley M-EE. Re-engage: a novel nurse-led program for survivors of childhood cancer who are disengaged from cancer-related care. J Natl Compr Cancer Netw. (2020) 18:1067–74. doi: 10.6004/jnccn.2020.7552, PMID: 32755982

[36] Michel G Mulder RL van der Pal HJH Skinner R Bárdi E Brown MC. Evidence-based recommendations for the organization of long-term follow-up care for childhood and adolescent cancer survivors: a report from the PanCareSurFup guidelines working group. J Cancer Surviv. (2019) 13:759–72. doi: 10.1007/s11764-019-00795-5, PMID: 31396878

[37] Wong CL Chan CWH Zhang M Cheung YT Chow KM Li CK. Care coordination models for transition and long-term follow-up among childhood cancer survivors: a scoping review. BMJ Open. (2024) 14:e087343. doi: 10.1136/bmjopen-2024-087343, PMID: 39160096 PMC11337698

[38] Jain J Qorri B Szewczuk MR. The crucial role of primary care providers in the long-term follow-up of adult survivors of childhood cancer. CMAR. (2019) 11:3411–8. doi: 10.2147/CMAR.S197644, PMID: 31118774 PMC6499444

[39] Demoor-Goldschmidt C Veillon P Esvan M Leonard M Chauvet S Bertrand A. A software tool to support follow-up care in a French childhood cancer cohort: construction and feasibility. BMC Cancer. (2024) 24:130. doi: 10.1186/s12885-024-11857-y, PMID: 38267891 PMC10809785

[40] Rosenberg AR Muriel AC. Poor mental health among survivors of childhood cancer-risk factors and a call for intervention. JAMA Pediatr. (2023) 177:758–9. doi: 10.1001/jamapediatrics.2023.2162, PMID: 37345507

[41] Deegan A Brennan C Gallagher P Lambert V Dunne S. Social support and childhood cancer survivors: a systematic review (2006-2022). Psycho-Oncology. (2023) 32:819–33. doi: 10.1002/pon.6128, PMID: 36944590

[42] Matsui M Taku K Tsutsumi R Ueno M Seto M Makimoto A. Role of peer support in posttraumatic growth among adolescent and young adult cancer patients and survivors. J Adolesc Young Adult Oncol. (2023) 12:503–11. doi: 10.1089/jayao.2022.0064, PMID: 36579948

[43] Yeom J-W Yeom I-S Park H-Y Lim S-H. Cultural factors affecting the self-care of cancer survivors: an integrative review. Eur J Oncol Nurs. (2022) 59:102165. doi: 10.1016/j.ejon.2022.102165, PMID: 35777220

